# Chronic exercise keeps working memory and inhibitory capacities fit

**DOI:** 10.3389/fnbeh.2014.00049

**Published:** 2014-03-11

**Authors:** Concepción Padilla, Laura Pérez, Pilar Andrés

**Affiliations:** ^1^Neuropsychology and Cognition group, Department of Psychology and Research Institute on Health Sciences, University of the Balearic IslandsPalma de Mallorca, Spain; ^2^Instituto de Investigación Sanitaria de PalmaPalma de Mallorca, Spain

**Keywords:** working memory, inhibition control, aerobic exercise, young adults

## Abstract

Padilla et al. (2013) recently showed that chronic aerobic exercise in young adults is associated with better inhibitory control as measured by the strategic Stop Signal Task (SST). The aim of the current study was to explore whether better inhibitory abilities, associated with high levels of physical fitness, were also associated with higher working memory capacity (WMC) in young healthy adults. Participants aged between 18 and 30 years and showing different levels of fitness confirmed by the Rockport 1-mile walking fitness test took part in this study. Active and passive participants were administered the SST to measure inhibitory control, and the Automatic Operation Span (AOSPAN) to measure verbal WMC. We first replicated Padilla et al.'s results showing that exercise specifically modulates strategic inhibitory processes. Our results also showed that active participants presented with better WMC than sedentary ones, showing a better capacity to manage simultaneously two verbal tasks and to inhibit interference. The results point to an association between chronic exercise, inhibitory abilities, and WMC. The theoretical relationship between these variables will be discussed.

## Introduction

Executive functions can be described as an umbrella term including a family of controlled (in opposition to automatic) processes, which can be separated in three core functions: working memory, inhibition and cognitive flexibility (Diamond, 2013). The conjunction of these functions allows carrying out more complex functions as reasoning, problem solving and planning.

Executive functions and one of its subcomponents – working memory capacity (WMC) – have been shown to be relevant in the efficient cognitive functioning and in the progression of several developmental and neuropsychological disorders, which has resulted in a pursuit of therapeutic ways to decelerate the deterioration of such capacities. This is the case of cognitive training (e.g., Klingberg, 2010) and cardiovascular activity (e.g., Colcombe et al., 2004; Weinstein et al., 2012), both based on the principle of neuroplasticity across the lifespan. In the case of cognitive training through computer programs, transfer to other tasks that are not directly trained has not yet been clearly demonstrated in any age group (Owen et al., 2010; Shipstead et al., 2012). However, cardiovascular exercise has shown its involvement in the improvement of a wide range of executive functions in children (Hillman et al., 2011), young (Padilla et al., 2013; Pérez et al., submitted), and older populations (Erickson and Kramer, 2009), which is believed to be mediated by the release of neurotrophic factors, such as brain-derived neurotrophic factor (BDNF), insulin-like growth factor type 1 (IGF-1) and vascular endothelial growth factor (VEGF). In turn these factors are associated with the increase of the temporal (Voss et al., 2013) and prefrontal lobes' (Colcombe et al., 2003, 2004, 2006) volume and connectivity. Furthermore, aerobic exercise has been related to an increment in brain vascularity in cortical areas and the hippocampus (Lopez-Lopez et al., 2004). Nevertheless, the effects obtained with both interventions, cognitive training and cardiovascular exercise, have not yet been demonstrated to be maintained in the long-term (Lustig et al., 2009).

Prefrontal areas and associated executive functions (Colcombe et al., 2004, 2006), seem more sensitive to the beneficial effect of exercise than other areas, as several studies with seniors, children, or clinical population have revealed (Tomporowski et al., 2008a; Hertzog et al., 2009; Davis et al., 2011; Chang et al., 2012). Even short-term aerobic exercise programs performed over a 6 months period by older populations have proven to exert an improvement in executive functions and increase in volume of some areas of the brain (Colcombe et al., 2006). However, in a recent review Guiney and Machado (2013) revealed that there is a lack of studies investigating the effects of aerobic exercise on a young cohort and the few that have been published have revealed mixed results. Differences among active and sedentary groups are found using evoked potentials, but not in behavioral data (Hillman et al., 2006; Themanson et al., 2006; Guiney and Machado, 2013).

Previous studies with young participants have mainly concentrated on the effects of acute exercise, the immediate effect of a range of intensive exercise like cycling or running carried out before or while the participant is doing the cognitive task (i.e., Themanson and Hillman, 2006; Huertas et al., 2011; see Guiney and Machado, 2013 for a review). The effects of this kind of exercise are temporary and not representative of the brain changes produced by long-term exercise. Besides, these studies do not control the level of exercise that participants carried out throughout childhood, which has been shown to exert an important influence in brain development (Chaddock, 2012). Etnier and Chang (2009) have also noted that previous studies have focused in a broad range of executive tasks, resulting in mixed results.

Arguably, well theoretically grounded tasks would enable to extract the specific processes affected by exercise. In addition, it is necessary to focus on the effects of chronic exercise compared to short-term exercise on cognition because it is more likely to produce permanent changes in the brain since it is undertaken following a long-lasting routine that will generate a protective cognitive reserve (Stern, 2009). Finally, it is important that the selection criteria for participants and the difference between active and passive participants in amounts of exercise and levels of fitness may also contribute to an effect of exercise on executive control. The higher the difference between active and passive participants in these variables, the more likely it will be to observe differences in cognition.

To address these problems, Padilla et al. (2013) investigated for the first time the effects of chronic exercise on executive functions in young adults using a highly reliable executive control task: the stop signal task (SST; Logan and Cowan, 1984; Verbruggen and Logan, 2008), which assesses motor inhibitory control associated with frontal lobe functions (Weinstein et al., 2012). In this case, participants were assessed using standard and strategic versions of the SST, and the results revealed better inhibitory abilities in active participants when the task was more executively demanding (strategic version). Pérez et al. (submitted) obtained similar results using the Attention Network test (ANT, Fan et al., 2002), with physically active participants revealing better performance in the executive network.

Trying to know the cause of these results, in the current study, we wondered to what extent these differences in inhibition control were related to a better WMC in physically active participants. WM is a system for temporarily storing and managing the information required to carry out complex cognitive tasks such as learning, reasoning, and comprehension. According to Kane and Engle (2002), WMC is a hierarchical system that consists of two components: executive-attention and short-term memory. These authors equate WMC to executive functions, making the differences between them blurred. They sustain that this system allows for the proper allocation of attentional resources and the active maintenance of the information needed to accomplish a goal-directed behavior or reasoning, avoiding at the same time the interference from other external stimuli or thoughts. In this online processing, several processes come into play; the storage and rehearsal of domain-specific information, as well as other executive functions (Conway et al., 2005) and controlled attention to sustain, divide and switch the focus of attention (Engle et al., 1999). A crucial point in this model is the relationship between WMC and inhibition. Engle and collaborators argue that inhibition and WMC correlate with each other, and Redick et al. (2007) suggest that WMC affects the ability to inhibit at any of the following stages: access, deletion or restraint (see Hasher et al., 1999 work for this distinction of inhibitory functions). In this vein, several studies using the extreme groups method (i.e., selecting the participants whose scores in a working memory task are under the 25th percentile and above the 75th percentile of the normal distribution), have shown that high WMC participants present with better inhibitory abilities than low WMC on tasks such as the flanker task (Redick and Engle, 2006), antisaccades (Unsworth et al., 2004) and proactive interference (Redick et al., 2007, 2011). Moreover, WM and inhibition have been associated to dorsal prefrontal cortex activation (Kane and Engle, 2002; Andrés, 2003).

Few studies have focused on the role that WMC could be playing in the associations between physical exercise and executive functions. In the case of young populations, Hansen et al. (2004) demonstrated that fitter young adults showed better accuracy in a 2-back task and Lambourne (2006) observed that active participants showed a higher WMC than passive participants in a reading span task. However, Kamijo et al. (2010) did not find better performance in a Stenberg task in fitter young adults compared to sedentary ones. Finally, it is important to note that none of these studies measured concurrently WMC and inhibition.

To this aim, we used Engle and colleagues' WM tasks, which involve the performance of two tasks at the same time. They require maintaining a variable number of items in mind while resolving complex problems. They are good predictors of performance on other higher level cognitive tasks, such as stroop or fluid intelligence tests; as well as disorders such as Alzheimer's, alcohol consumption or stress management (Engle et al., 1999; Unsworth et al., 2005). It has been shown that WM tasks have high reliability and validity (Conway et al., 2002). In the case of the Automatic Operation Span Task (AOSPAN; Unsworth et al., 2005), it measures the phonological loop and the central executive component of WM, which is highly associated with controlled processing and attention (Baddeley, 1986, 1996).

In the present study we investigated three hypotheses. First, we wanted to replicate the results observed in our previous study (Padilla et al., 2013) showing better inhibitory abilities in physically active participants using the strategic version of the SST, which makes greater demands on executive resources as will be explained in the method section. Second we predicted that aerobic exercise would enhance WMC. Third, we evaluated to what extent the active group's better inhibitory control could be linked to a greater WMC, as suggested by Kane and Engle (2002). Thus, we expected a high relationship between the inhibitory control showed under the strategic instructions, and the WMC. A wider WMC should be associated to a better ability to inhibit interference.

The results confirmed the advantage in inhibition of active participants previously observed (Padilla et al., 2013), i.e., the physically active group showed a speeded inhibitory response when strategic instructions were applied, but most importantly, the whole active group exhibited a greater WMC. The possible relationship between these variables will be discussed.

## Methods

### Participants

Fifty eight participants ranged between 18 and 30 years of age (*M* = 22.26, *SD* = 3.26) were assigned to the active or passive groups according to their fitness levels (see Table 1 for demographic details). The active group was formed by 29 participants with an average age of 22.21 (*SD* = 3.28), while the passive group consisted of 29 individuals with an average age of 22.31 (*SD* = 3.29). Each of these groups were further subdivided into standard (*n* = 14) and strategic (*n* = 15) subgroups according to the version of the SST that they performed. Participants were allocated to the active group if they had been doing aerobic exercise for at least 10 years, following a minimum routine of 6 h per week, distributed across at least 3 days a week. On the other hand, participants were allocated to the passive group if they had not been exercising for the last 4 years more than 1 h per week. The type of exercise that had been practiced during the last 4 years by the passive group could not have been cardiovascular (e.g., yoga, stretching, etc. were allowed). Also, they should not have done more than 6 h per week of aerobic exercise during their childhood (from 0 to 12 years old). This criterion was applied taking into account the fact that children in Spanish schools have at least 3 h per week of physical education.

**Table 1:** **Demographic variables**.

	**Active**		**Passive**	
	**Strategic**	**Standard**	**Strategic**	**Standard**
*n*	14	15	14	15
Age	22.71 (3.49)	21.73 (3.11)	21.14 (3.09)	23.40 (3.20)
Education	15.36 (3.41)	14.13 (3.68)	14.57 (3.98)	13.13 (1.89)
Vocabulary	42.29 (9.12)	43.40 (3.78)	42.79 (7.31)	48.33 (5.09)
Rockport^*^	57.82 (7.04)	57.24 (9.71)	47.26 (5.93)	44.43 (9.72)

*Average and SDs (in brackets) for Age (age of participants at the moment of testing), Education (number of completed years of formal education), Vocabulary (WAIS' Vocabulary subtest score) and Rockport test (Rockport Fitness Walking Test score)*.

* *Effect at p < 0.001*.

Before the participants started the testing, they were interviewed by telephone following a questionnaire about demographic data, lifelong exercise routines, medical history and education. If they fulfilled the requirements to participate, they were invited to come to our university facilities to perform the testing in a 2 h session. All participants gave their informed consent and were paid or given course credits if they were students. The experiment was performed in accordance with the ethical standards stated in the 1964 Declaration of Helsinki. Each activity group was subdivided in two other groups depending on the SST instructions they received: strategic or standard.

### Cardiorespiratory capacity

As in Padilla et al.'s (2013) study, maximal oxygen uptake was measured with the Rockport 1-mile Fitness Walking Test (Kline et al., 1987). This test was chosen due to its high correlation coefficient (0.88) with a direct index of VO_2_max, carried out using a treadmill (Kline et al., 1987; Weiglein et al., 2011). VO_2_ max is the maximal oxygen uptake that the organism is able to consume when it is carrying out a sub-maximal exercise. The higher the score, the higher the aerobic capacity and oxygen uptakes.

### Design and procedure

First, a telephone interview was carried out to gather information about the demographic data of each participant. They were asked about their level of education, and medical history to exclude participants who suffered or had suffered in the past from any mental disorder or physical illness that could affect the results. In addition, they were asked about the frequency of exercise they had done along their whole life. If they met the criteria to participate, they were invited to come to our facilities to take part in our study.

In a 2 h session, participants completed a more detailed health questionnaire with an experienced clinical psychologist, where they had to specify whether they were having any mental or physical problem and/or taking any medication at that time. After that, they carried out the SST and AOSPAN tasks in a quiet room. Later, they completed the Wechsler Adult Intelligence Scale Vocabulary subtest and finally they completed the Rockport 1-mile Fitness Walking Test on the University campus, where they had to walk 1 mile as fast as possible to measure their initial and final pulse and the time they took to complete the distance.

#### SST task

The SST (Verbruggen et al., 2008; Padilla et al., 2013) task was presented on a LG computer with a 19″ Phillips monitor with a resolution of 1024 × 768 pixels. The task was programmed using the E-prime software (Schneider et al., 2002). Participants were seated approximately at 50 cm of distance from the screen and wore headphones.

**Figure 1:**
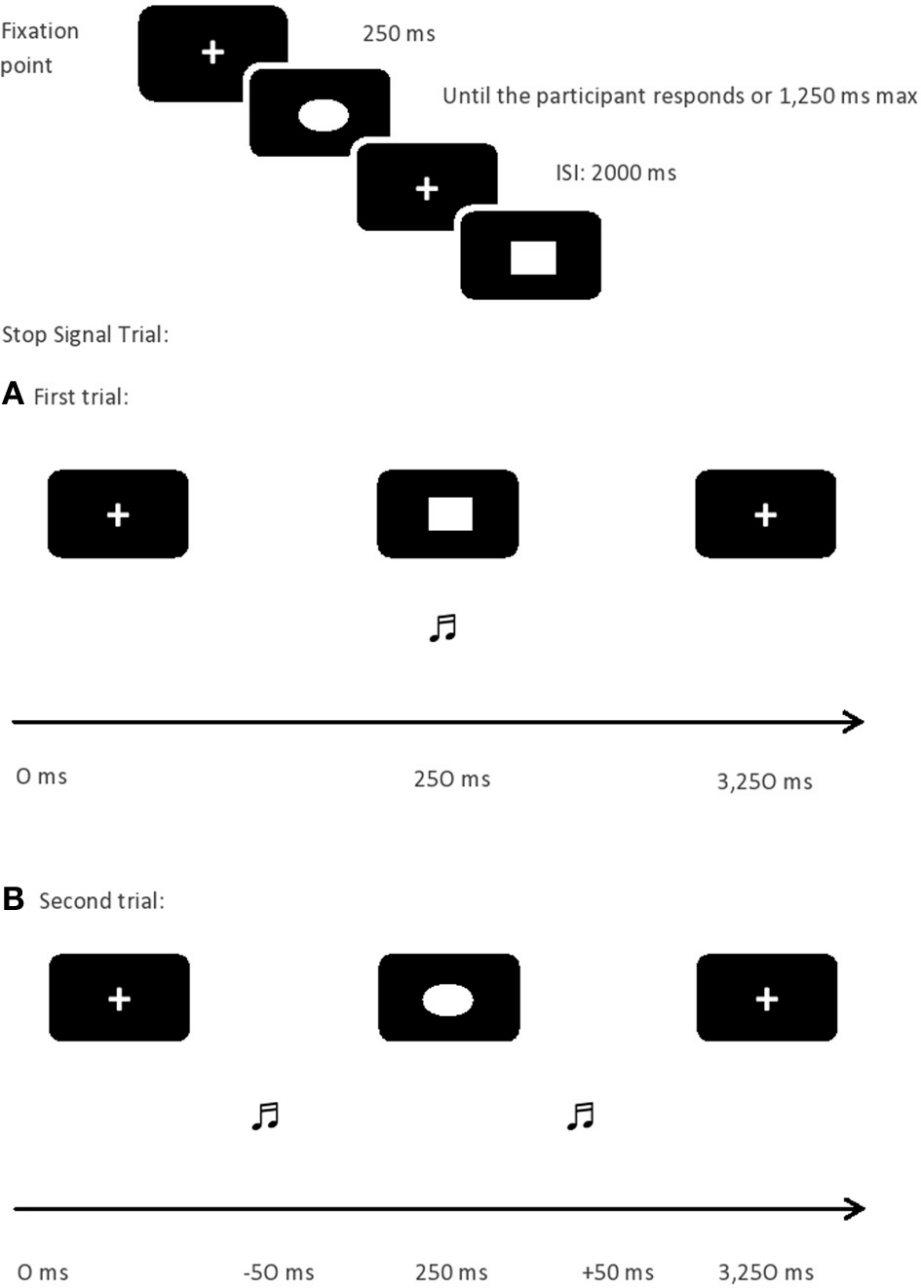
**Stop Signal Task design**. Note: ISI, Inter Stimulus Interval; ms, milliseconds.

As can be seen in Verbruggen et al. (2008), SST begins with the appearance of a fixation sign (+) followed by a stimulus drawn in white color presented in the center of a screen in black. Two types of trials were presented at random: the GO (75%) and the STOP (25%) trials. In the GO trials, participants had to decide as fast as possible whether a geometric figure displayed on the screen was a square or a circle. They responded by pressing “Z” or “−” on the keyboard with the index fingers. In the STOP trials, the procedure was the same with the difference that a tone was presented shortly after the geometric figure, and participants had then to inhibit their response. The interval between the geometric figure and the STOP signal followed a tracking procedure: when participants successfully withheld a response in a STOP trial, the interval between the figure and the stop signal was incremented by 50 ms; however, when participants failed to withhold their response, it was decreased by 50 ms. Doing so, the probability to inhibit a response is random, thereby, there is a 50% of likelihood of correctly withdrawing a response (see Figure 1).

The assessment procedure was the same as in Padilla et al.'s (2013), maintaining the same task conditions: standard and strategic. Both tasks were similar; the only difference between them is the instructions. In the standard condition (Verbruggen et al., 2008), participants were told that on the 25% of trials a tone was going to be presented. For half of these trials, it would appear very early and it would be relatively easy to withhold a response. On the other half of the STOP trials, the tone would come late, increasing the difficulty to inhibit the response. Importantly, participants were also warned that they should not postpone the responses while waiting for the potential occurrence of the stop signal. However, in the strategic condition participants were just told how to respond to the stimulus that would appear on the screen, and asked to withdraw their response when a sound appeared. They were asked not to wait to know whether the sound would appear or not, allowing them to apply the strategies they decided.

The variables measured by this test were: (a) the go RT: time to respond to the go trials; (b) the stop signal delay (SSD): mean delay between visual and auditive stimuli along all stop signal trials; (c) the stop signal RT (SSRT): latency of the inhibition process calculated by subtracting the mean SSD from the mean RT in go trials; (d) the signal respond RT (SRRT): mean time to respond incorrectly in the stop trials; (e) the percentage of correct responses in the go trials; and (f) the percentage of missed responses in the go trials.

#### Automatic operation span

AOSPAN (Unsworth et al., 2005) began with three blocks of practice. First, the letter practice block trained the participant to remember different sets of letters that could contain from 2 to 5 letters. Once the participants had completed a set, 12 letters were displayed in a matrix of 12 and they were asked to mark in order the letters that had been shown previously. After that, a feedback message was shown to inform participants about the number of letters correctly remembered. The second block was to practice solving a series of additions, subtractions, multiplications or divisions of one digit numbers as fast as possible. In this block, participants were presented with the operation, then had to click on the mouse left button once they had the solution in mind and then, a screen with a number appeared, in which the participant had to decide whether that number was the right solution to the problem (“false” or “true”). During this block, the software calculates the averaged time to solve these operations to set the maximum exposition time of the operation task in the experimental block. In the third practice block, both tasks were combined as with the experimental block. Individuals had first to remember in order the letters presented and then solve the arithmetical problems as fast as possible. This was followed by a variable number of trials, from two to five. Finally, participants had to say in order the letters that they remembered. The experimental block was similar to the last practice block, but the time to solve the arithmetic problem was limited to the averaged time calculated in the second practice block. The dependent variable was the total number of letters correctly recalled in all sets. This measure reflects WM *capacity* relatively uncontaminated by the processes involved in serial recall, which tend to be executive.

## Results

The resulting four groups of participants did not differ in terms of age [*F*_(1, 54)_ = 0.003, *MSE* = 0.033, *p* = 0.955, η^2^_*p*_ = 0.000] or years of formal education [*F*_(1, 54)_ = 1.046, *MSE* = 11.546, *p* = 0.311, η^2^_*p*_ = 0.019] (see Table 1). They showed similar vocabulary levels measured with the Vocabulary subtest from the Wechsler Adult Intelligence Scale (Wechsler, 1999), [*F*_(1, 54)_ = 2.469, *MSE* = 106.887, *p* = 0.122, η^2^_*p*_ = 0.044]. Active participants had practiced cardiovascular exercise for an average of 204.621 months (17.052 years) and a total of 8488.107 h (*SD* = 5008.938) during their lives. The passive participants had practiced aerobic exercise during their lives, that is, before the past last 4 years and mostly during their childhood, which we consider from 0 to 12 years old; in an average of 67.414 months (5.618 years) with a mean of 1559.079 h (*SD* = 1646.452) across the lifespan. Averages of months [*F*_(1, 54)_ = 70.575, *MSE* = 269507.589, *p* < 0.001, η^2^_*p*_ = 0.567] and hours of sport [*F*_(1, 53)_ = 46.882, *MSE* = 680356813.058, *p* < 0.001, η^2^_*p*_ = 0.469] were significantly different between passive and active participants.

As expected, physically active participants showed higher scores than passive in the Rockport test: 57.517 (*SD* = 8.381) against 45.795 (*SD* = 8.100) [*F*_(1, 54)_ = 28.515, *MSE* = 1976.772, *p* < 0.001, η^2^_*p*_ = 0.346], which means active participants presented higher cardiovascular capacity as a consequence of their exercise routines.

**Figure 2:**
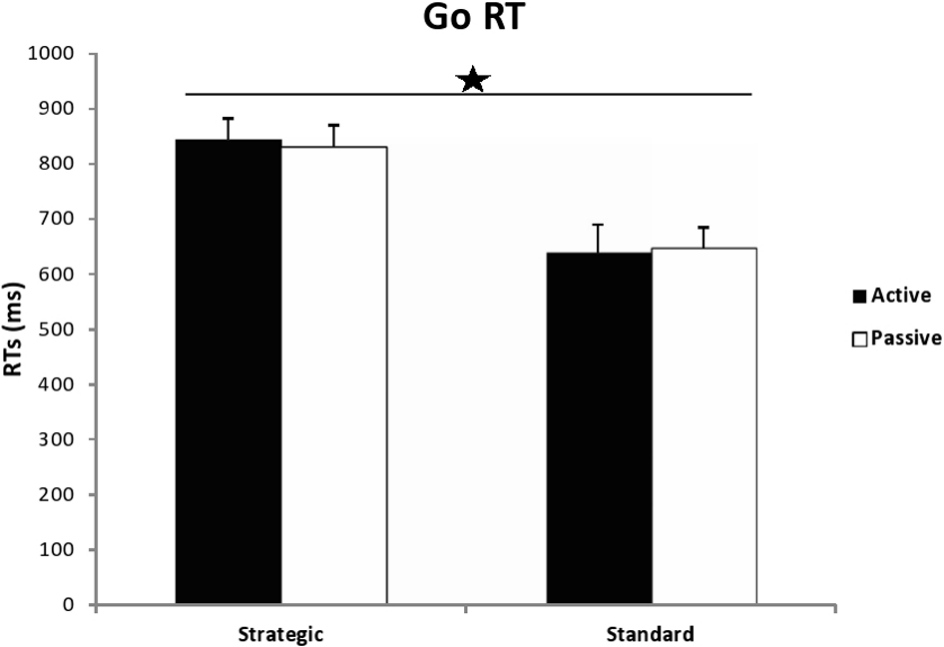
**Go RTs in milliseconds for active and passive participants in both conditions of the Stop Signal Task: strategic and standard**. The star indicates significance (instruction effect) with *p* < 0.05.

**Figure 3:**
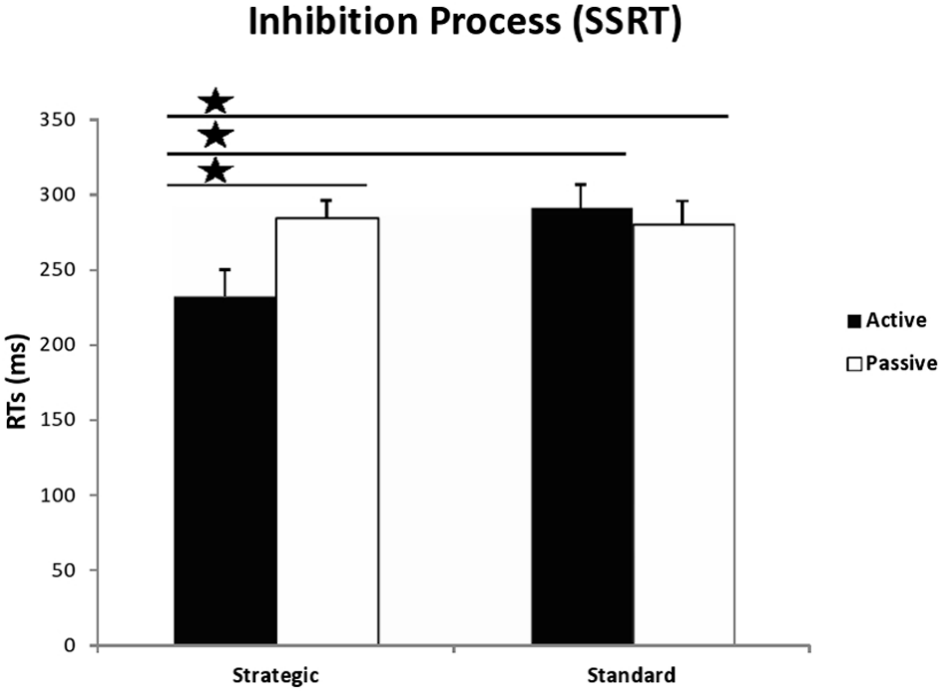
**Latency of the inhibition process (SSRTs) in milliseconds, calculated by subtracting the mean SSD from the mean RT in go trials, for active and passive participants in both conditions of the Stop Signal Task: strategic and standard**. The stars indicate significant differences between strategic active group and the remaining groups with *p* < 0.05.

The most important measures of the SST, which were used to test our hypotheses, were GO RTs (the time taken to respond to the primary or go task) and SSRT (the time required to inhibit an already initiated response, that is, inhibition control). These measures are presented in Figures 2, 3, while all other measures (SSD, SRRT, percentages of correct and missed responses) are reported in Table 2.

**Table 2:** **Stop signal task variables**.

	**Active**		**Passive**	
	**Strategic**	**Standard**	**Strategic**	**Standard**
SSD^*^	611.91 (175.30)	348.98 (181.84)	547.28 (157.70)	368.43 (150.03)
SRRT^*^	752.49 (165.76)	569.67 (167.42)	743.96 (136.37)	545.43 (112.29)
Go Accuracy^*^	90.94 (11.34)	97.55 (3.24)	90.06 (12.89)	98.32 (1.67)
Go miss^*^	8.46 (11.48)	1.79 (3.44)	9.34 (13.12)	0.89 (1.20)
Go error	0.60 (1.02)	0.65 (1.11)	0.60 (0.91)	0.79 (0.87)
Stop accuracy	48.36 (6.63)	52.10 (5.19)	50.01 (8.36)	50.05 (5.55)

Average and SDs (in brackets) for SSD, Delay between visual and auditive stimulus; SSRT, Inhibition process latency; SRRT, RT incorrect responses in the stop trials; Go Accuracy, % correct responses in Go trials; Go Miss, Miss responses in Go trials; Go Error, response errors in Go trials; and Stop accuracy, correct inhibited responses in stop trials;

* *Effect of instructions at p < 0.005*.

A univariate 2 (group) × 2(instructions) ANOVA carried out on the GO RTs, revealed a significant effect of instructions [*F*_(1, 54)_ = 22.407, *MSE* = 546152.009, *p* < 0.001, η^2^_*p*_ = 0.293], whereby the strategic version yielded longer RTs (*M* = 838.678, *SD* = 140.506) than the standard one (*M* = 644.487, *SD* = 164.513). No significant effect of group [*F*_(1, 54)_ = 0.004, *MSE* = 107.863, *p* = 0.947, η^2^_*p*_ = 0.000] or instructions × group interaction [*F*_(1, 54)_ = 0.066, *MSE* = 1609.640, *p* = 0.798, η^2^_*p*_ = 0.001] were found.

A univariate 2 (group) × 2(instructions) ANOVA on the SSRT data revealed a trend for the effect of instruction [*F*_(1, 54)_ = 3.190, *MSE* = 10824.943, *p* = 0.080, η^2^_*p*_ = 0.056], and, most importantly, a significant instruction × group interaction [*F*_(1, 54)_ = 4.227, *MSE* = 14344.243, *p* = 0.045, η^2^_*p*_ = 0.073]. As in Padilla et al.'s (2013) study, *t*-tests revealed that active participants exhibited faster SSRT than passive participants in the strategic [*t*_(26)_ = −2.460, *p* = 0.021, *d* = −0.965], but not the standard condition [*t*_(28)_ = 0.488, *p* = 0.630, *d* = 0.184]. Furthermore, active participants inhibited responses faster under strategic instructions compared to standard instructions [*t*_(27)_ = −2.489, *p* = 0.019, *d* = −0.958], while passive participants, in contrast, showed similar SSRTs regardless of instructions [*t*_(27)_ = 0.212, *p* = 0.834, *d* = 0.082]. Finally, the comparison between SSRTs from the active participants in the strategic condition and the passive participants in the standard condition was just significant [*t*_(27)_ = −2.057, *p* = 0.050, *d* = −0.79]. In sum, active participants from the strategic condition presented with better inhibitory responses than the remaining groups, as can be seen in Figure 3.

Further univariate 2 (group) × 2 (instructions) ANOVAs were carried out on the remaining measures (SSD, SRRT, percentage of correct responses, and missed responses). They all revealed main effects of instructions (all *p*s < 0.005), but no significant effect of group or group × instructions interaction (all *p*s > 0.341). It is noteworthy that the number of responded trials decreased in the strategic version, but the number of errors remained the same between standard and strategic conditions (see Table 2).

**Figure 4:**
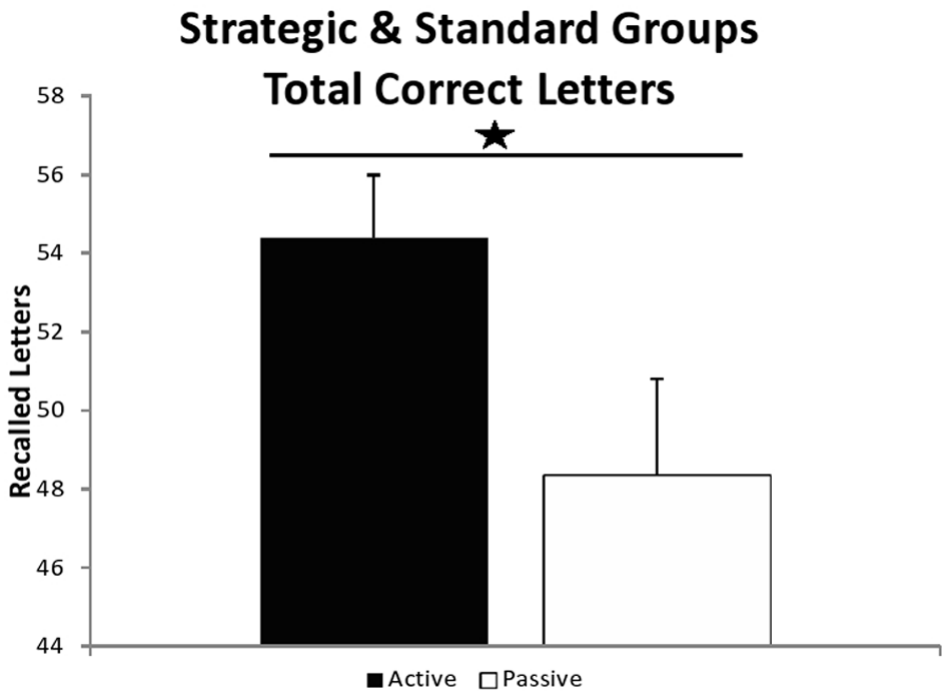
**Recalled correct letters**. Recalled correct letters in the whole sample (strategic and standard groups). ^*^Effect at *p* = 0.042.

Regarding WMC (Figure 4), a univariate 2 (group) × 2 (SST condition: strategic vs. standard) ANOVA showed a significant group effect [*F*_(1, 54)_ = 4.309, *MSE* = 539.745, *p* = 0.043, η^2^_*p*_ = 0.074], revealing greater WMC for the active (*M* = 54.414, *SD* = 8.471) than for the passive participants (*M* = 48.379, *SD* = 13.116) [*t*_(56)_ = 2.081, *p* = 0.042, *d* = 0.556]. There was no SST condition × group interaction [*F*_(1, 54)_ = 0.480, *MSE* = 60.159, *p* = 0.491, η^2^_*p*_ = 0.009]. There were no differences among SST conditions, participants who belonged to the strategic group, showed a similar WMC than those who belonged to the standard group [*F*_(1, 54)_ = 0.013, *MSE* = 1.656, *p* = 0.909, η^2^_*p*_ = 0.000]. A separated ANOVA was performed with just the participants that carried out the strategic version, finding here also significant differences [*F*_(1, 26)_ = 4.254, *MSE* = 464.143, *p* = 0.049, η^2^_*p*_ = 0.141].

Once we observed a greater WMC in the physically active group, we ran an ANCOVA to evaluate the extent to which WMC could explain the differences between active and passive participants observed in the SSRT scores. The results showed that the instruction × group interaction [*F*_(1, 53)_ = 3.754, *MSE* = 12578.461, *p* = 0.058, η^2^_*p*_ = 0.066] did no longer reach statistical significance after controlling for WMC, indicating that WM capacity and SSRT scores were related to some extent. When the ANCOVA was applied only to the sample from the strategic version, the group effect did not reach statistical significance either [*F*_(1, 25)_ = 4.162, *MSE* = 13485.547, *p* = 0.052, η^2^_*p*_ = 0.143].

Finally, the relation between exercise, WMC and inhibition (SSRT in the strategic condition) was evaluated with a hierarchical multiple regression analysis that entered group (active and passive) as the predictor variable in the step 1, and WM capacity in the step 2 to evaluate its additional contribution. Simple correlation values of all pairs of variables are shown in Table 3. The *R square* in step 1 was 0.189, which was highly significant [*F*_(1, 26)_ = 6.054, *MS*_residual_ = 3155.189, *p* = 0.021], indicating a relationship between exercise and inhibition. However, the *R*^2^ change in step 2 was 0.010, which was not significant [*F*_(2, 25)_ = 3.105, *MS*_residual_ = 3240.495, *p* = 0.062], indicating no significant relationship between inhibition and WM.

**Table 3:** **Correlations found between inhibition and multiple variables**.

**Correlations**								
		**Age**	**Total months**	**Total hours**	**Rockport**	**Wais**	**Education**	**Total correct letters**
*r*	SSRT	−0.11	−0.45	−0.19	−0.20	−0.06	0.08	−0.26
*p*	SSRT	0.59	0.02	0.34	0.31	0.76	0.69	0.19

*R is the Pearson Correlation and p is the p-value indicating the level of significance*.

## Discussion

The aim of this study was to investigate inhibitory/executive control and WMC in physically active compared to passive participants. To this aim, we used the SST (Verbruggen et al., 2008; Padilla et al., 2013) and the AOSPAN task (Unsworth et al., 2005) to evaluate inhibition and WMC respectively. We also investigated to what extent the group differences observed in inhibition/executive control could be related to WMC.

Our results were in line with expectations in regard to the effect of task manipulations on performance: the strategic version of the SST gave rise to longer GO RTs than the standard version, replicating Padilla et al.'s (2013) findings. These results confirm that the strategic and standard versions of the SST are measuring different ways to deal with the task, with the strategic one allowing for the implementation of the “goal priority strategy” (Leotti and Wager, 2010; Sella et al., 2013), which consists of the lengthening of the GO RTs in order to improve the performance in the stop signal trials. When instructions allowed participants to apply a trade-off as in the strategic version, SST was analogous to a dual task, where each task must be carried out at the same level of accuracy and speed. The cognitive resources must be divided to control the performance in both tasks. The consequences of “the goal priority trade-off” are that participants wait longer to produce their response in the go trials to make sure the stop signal is not going to appear. This gives rise to an increased number of omissions, as participants produce their response after the maximal interval they are allowed to respond (see Table 2). This pattern of results reflects a different (more executive) way to deal with the task in its strategic version (although the different instructions did not affect the stop accuracy or the number of errors in the go trials).

Second, the results replicated Padilla et al.'s (2013) in showing that the physically active participants obtained a better inhibitory control (shorter SSRTs) than the passive ones, but only under the strategic condition of the SST. It is important to note that both groups of participants in the strategic condition had the same number of errors than the standard condition groups, but just the active participants in the strategic condition were faster withdrawing their responses in the stop signal trials compared to the remaining groups. Also, active participants were faster inhibiting their responses in the strategic version of the task than active participants in the standard condition, but this difference was not observed in passive participants. This is consistent with the findings by Pérez et al. (submitted) showing a relatively specific relationship between exercise and executive attention when using the ANT task (Fan et al., 2002). Since active and passive participants showed similar go RTs within each version of the SST (strategic and standard), it is important to note that both groups did not differ in terms of general speed of processing (Salthouse, 1996), which means that the benefit induced by chronic exercise is specific to the inhibition process.

Third, the results also revealed higher WMC in active compared to passive participants as we expected. A recent review studying the effects of acute aerobic exercise on working memory in young adults (Verburgh et al., 2013) revealed a very low effect size (*d* = 0.05), however, we obtained a medium effect size (*d* = 0.556). Importantly, the results showed that controlling for WMC as a covariate, reduced the group differences in inhibition to the point that the group effect on inhibition (active group in the strategic condition) no longer reached statistical significance.

However, although WMC explains a percentage of inhibition variance (strategic SSRT), this did not result in a strong relationship, since the regression between WMC and SSRT did not reach significance. It could be argued that this might be linked to the size of our sample. Nonetheless, using a significantly bigger sample (*n* = 262), Wilhelm et al. (2013) did not find correlations either between tasks that assess inhibition and interference control, such as Flanker and Simon tasks, and those that evaluate WM, although they did find a high correlation between updating, complex-span and binding tasks. One of the possible explanations that Wilhelm et al. (2013) raised about Engle's group findings showing correlations between WMC and inhibition is the use of the extreme-groups method, which removes most of the variability from the group, increasing in turn the likelihood to find correlations between WM and inhibition (Preacher et al., 2005). Here, it is worth mentioning that other studies that did not apply the extreme-groups technique did not find correlations between WM and inhibition (Friedman and Miyake, 2004; Hofmann et al., 2009). It is therefore likely that inhibition and WMC show some processing overlap and support each other, but they are also independent and none seems to be the unique cause of the other.

Our results are consistent with the suggestion by Davidson et al. (2006) and Zanto et al. (2011) claiming that WMC and inhibition, although independent, are interrelated and work together. These authors suggest that WM supports inhibitory control holding the task goal in mind. Focusing on the task decreases the probability of interference from irrelevant stimuli. At the same time, inhibitory control supports WM in different ways, for example, preventing recovery of related but unwanted memories, or avoiding the emergence of distractors. If this kind of information is not inhibited, it may result in mind-wandering (Diamond, 2013).

Roberts and Pennington (1996; also see Nyberg et al., 2009) attempted to understand the interaction between WM and inhibition attending to the premise that they are independent processes sharing limited resources. They suggest that inhibitory performance results from a dynamic interaction among one's WMC, the strength of the competing prepotencies or inhibitory task demands, and the WM task demands. It is only when demands on inhibition and/or WM reach high enough levels that this competition for common limited resources, or resource sharing, takes place. The individual differences in capability will therefore only be observed when the task demands are high (when they exceed a certain threshold). The implication of this is that “tasks that require both (WM and inhibition) are more likely to tax the PFC, although tasks that have a very high demand for either are also hypothesized to be prefrontal tasks” (Pennington and Ozonoff, 1996, p. 338).

The pattern of results observed in our studies is well explained under this model. We did not find any difference between active and passive participants in the standard condition of the SST (nor did we in Padilla et al.'s 2013 study), given that the attentional and WM demands are relatively low. However, the groups differed in the more executive version of the SST, where the physically active group showed better inhibition control. That the standard version of the SST does not require great deal of attentional or WM resources is supported by the finding by Yamaguchi et al. (2012) that response inhibition does not suffer from dual task interference. Since active participants present with higher WMC, this enables them to deal with the higher WM demands of the strategic version of the SST. However, when little WMC is required by the task, as it is the case in the standard version of the SST, the higher WMC observed in the active participants does not come as an advantage to contribute to the inhibitory process, resulting in no differences in inhibition between active and passive participants.

We emphasize that in our study we focused on the effect of aerobic chronic exercise as opposed to aerobic acute exercise as examined in past studies with young adults (e.g., Huertas et al., 2011). There are few studies studying the effects of chronic exercise in this age group (Verburgh et al., 2013), given that cognitive functions at this period of life are at their maximum level (Salthouse and Davis, 2006), and they are less likely to be affected by exercise interventions as a ceiling effect may be observed. Most of the studies with young adults have failed to demonstrate differences among active and passive participants with behavioral data. Those that have found an effect of exercise have used psychophysiological measures, demonstrating different patterns of brain activation. For instance, Hillman et al. (2006) and Themanson and Hillman (2006) revealed differences in the P3 component between young active and passive adults using the task-switching paradigm. Themanson and Hillman (2006) and Themanson et al. (2006) showed a lower error related negativity amplitude (ERN or Ne) and a higher error positivity (Pe) in the active group. Nevertheless, further studies using neuroimaging techniques are necessary to elucidate the positive effects of aerobic exercise on brain structure and connectivity in this group of age. For this reason, we used a strict selection criterion for the recruitment of participants, with active participants having practiced aerobic exercise for at least 10 years with a frequency of at least 6 h a week. We also decided to apply a more strategic task to deal with the likelihood of a ceiling effect.

Acute and chronic exercise has different effects on the brain. Acute exercise spans from 10 to 40 min and the cognitive tasks may be applied during or after the aerobic exercise is being performed. Chronic exercise, instead, range from periods of training of 3–6 weeks (Griffin et al., 2011), 6 months or 1 year, up to 10 years in our case. Acute exercise is related to an increase in brain blood flow, as well as the levels of vasopressin, β-endorphine, catecholamines, and adrenocorticotropic hormone in plasma (Chmura et al., 1994; McMorris et al., 2008), which is thought to reflect neurotransmitters levels in the brain and lead to an elevated arousal that would enhance cognitive performance. A recent meta-analysis (Verburgh et al., 2013) has found a moderate positive effect of acute exercise (*d* = 0.52) on executive functions in children, adolescents, and young adults, that was more pronounced in inhibition/control processes than working memory tasks. It is worth noting to remark that these effects are temporary, since the tasks are applied during or just before participants are doing the exercise. On the contrary, chronic exercise is accompanied by more permanent physiological changes in the brain, such as the formation and extension of new vessels, which result in the improvement of brain perfusion. Also, neurogenesis and release of neurotrophic factors take place increasing the chances of neural growth and survival, which affects learning and memory learning capabilities (Voss et al., 2011). Larger brain volumes have been shown in active children (Chaddock et al., 2011) and old adults (Colcombe et al., 2006), while there is a lack of research using neuroimaging in young adults. Thereby, it is more likely to induce brain cognitive reserve with chronic compared to acute exercise (Ahlskog et al., 2011; Smith et al., 2013), as several studies with healthy children or old adults have suggested (Tomporowski et al., 2008b; Howie and Pate, 2012; Voss et al., 2013). These interventions have promising results for combating mental disorders such attention deficit hyperactivity disorder (ADHD) (Gapin et al., 2011) or dementia (Ahlskog et al., 2011; Smith et al., 2013).

Regarding the cognitive processes measured in our study, previous results (see Hillman et al., 2008 for a review) have shown that long-term high cardiovascular fitness gives rise to significant volumetric and functional improvements particularly in prefrontal areas, which underpin inhibition and executive processes. Recent research has revealed for example that gray matter volume of the right inferior frontal gyrus mediates the relationship between higher cardiovascular fitness and interference control in the Stroop task in older adults (Weinstein et al., 2012). It is therefore possible that the functional network that supports inhibitory mechanisms is preferentially boosted by the cardiovascular effects of exercise, which is consistent with the pattern of results observed in our studies revealing a relatively specific effect of chronic exercise on tasks requiring inhibitory control (Padilla et al., 2013; Pérez et al., submitted).

The results from the current study show that inhibition and WM can be potentiated by the chronic practice of physical exercise, which can be defined as a kind of exercise performed under a high frequency and long-term routine; in comparison with individuals who have a very sedentary lifestyle.

Our results also suggest that inhibition and WM are independent processes, but dependent on a limited shared capacity. This capacity is the quantity of information that can be held active, and that makes us self-aware. WM and inhibition processes are necessary to carry out a goal-directed task. However, in most cases, inhibition processes depend on WM, since it is crucial to keep in mind what must be inhibited (executive processes are “superordinate” in relation to inhibition, Nyberg et al., 2009).

Concerning our experimental design, cross-sectional studies that explore the influence of long-term aerobic exercise on cognition, brain function, and structure, along with cognitive reserve would be necessary in future studies. Most of the studies that are carried out under the category of chronic exercise do not span the range of more than 1 year and do not explore the effects once the intervention has finished. The present study is a better way to evaluate how exercise gives rise to cognitive reserve, since it accounts for the true chronic exercise that is performed throughout life, although not all variables can be controlled for.

Future research should establish how different ranges of physical activity in terms of frequency and years of aerobic exercise can affect cognitive performance, brain volume or connectivity, instead of being chosen arbitrarily. For example, it can be differentiated between acute, short-term, and long-term interventions.

Moreover, more executive tasks are recommended to challenge executive functions in a way that inhibition and WMC demands are high enough to see the benefits of exercise in young populations, as we have demonstrated in our study. Neuroimaging studies would also be required to establish the functional and structural brain changes produced by chronic aerobic exercise in young populations.

Finally and to conclude, the present study has demonstrated that chronic aerobic exercise benefits not only physical, but also cognitive functions across the lifespan.

## Author contributions

Pilar Andrés and Concepción Padilla designed and planned the study, analyzed, and interpreted the data and wrote the manuscript. Laura Pérez collaborated in the design and planning of the study and helped in data collection.

### Conflict of interest statement

The authors declare that the research was conducted in the absence of any commercial or financial relationships that could be construed as a potential conflict of interest.
